# The Principles and Practice of Surgery

**Published:** 1873-09

**Authors:** 


*The Principles and Practice of Surgery.* By Frank Hastings Hamilton, A. M., M. D., LL. D., etc. Illustrated with 467 Engravings on wood. Second Edition, Revised and Corrected. New York: Wm. Wood & Co. 1873.

It is so short a time since we had occasion to notice the first edition of Prof. Hamilton's work that it is almost unnecessay to say anything further than that a new edition has been issued. The typographical errors of the first edition have been corrected, a few modifications made in certain statements made in the first edition, and a few important facts have been added. The index has also been greatly extended, which will be recognized by the readers of the book as a great improvement.

Among the additions which have been made we notice a mention of the mode of evacuating the bladder by Deaulafoy's Aspirator, and a short paragraph on Renal Calculi. Other new and important observations have been noted in various portions of the work. The improvements are all of much value, and the new edition presents many advantages over the former one. As a text book for students it is equal to any, and we can heartily recommend it to both students and practitioners.

